# Intraoperative OCT-guided partial internal limiting membrane peeling combined with inferior flap coverage for the treatment of idiopathic macular holes: A prospective randomized controlled clinical trial

**DOI:** 10.1097/MD.0000000000045392

**Published:** 2025-11-07

**Authors:** Jinghai Mao, Wendie Li, Yanyan Wang, Sangsang Wang

**Affiliations:** a Department of Ophthalmology, Ningbo Eye Hospital, Ningbo, China.

**Keywords:** idiopathic macular holes, ILM peeling, intraoperative OCT, surgical outcomes, visual acuity

## Abstract

To evaluate the efficacy and safety of intraoperative optical coherence tomography (iOCT)-guided partial internal limiting membrane (ILM) peeling combined with inferior flap coverage for the treatment of idiopathic macular holes (IMH). This prospective, randomized, controlled clinical trial included patients with Gass stage 4 IMH, aged 50 or older, who had not undergone prior vitreoretinal surgery. Patients were randomized into 2 groups: the experimental group received iOCT-guided partial ILM peeling with inferior flap coverage, and the control group underwent standard ILM peeling. Outcome measures included best-corrected visual acuity, optical coherence tomography (OCT), optical coherence tomography angiography (OCTA), multifocal electroretinography, and microperimetry at 2 weeks and 6 months postoperatively. A total of 34 eyes were enrolled, with 18 in the experimental group and 16 in the control group. There was a significant improvement in best-corrected visual acuity and reduction in central foveal thickness in both groups, with no statistically significant differences between groups at any time point. The hole closure rate was also comparable, with 83.3% in the experimental group and 75.0% in the control group at 2 weeks, and 94.4% and 87.5% at 6 months, respectively. Functional outcomes, as measured by OCTA, microperimetry, and multifocal electroretinography, showed significant improvements in both groups without significant intergroup differences. iOCT-guided partial ILM peeling with inferior flap coverage is a feasible and safe surgical approach for IMH, with outcomes not statistically different from the standard ILM peeling technique. The novel technique may offer subtle benefits in preserving retinal structure and function, warranting further investigation.

## 1. Introduction

Idiopathic macular holes (IMH) are a significant cause of visual impairment, particularly among the elderly population, and are characterized by the presence of a full-thickness defect in the fovea, leading to central vision loss and metamorphopsia.^[1]^ The pathogenesis of IMH is multifactorial, with vitreomacular traction being a key factor in its development.^[2]^ Given that patients with IMH experience a significant decrease in visual acuity, often accompanied by metamorphopsia and central scotoma, which severely impairs their quality of life, the prognosis for visual function remains guarded even following successful surgical closure of the hole. Therefore, enhancing the hole closure rate and maximizing the restoration of visual function have become critical objectives for ophthalmologists.^[3]^ Over the past few decades, there have been significant advances in the surgical management of IMH, with the introduction of pars plana vitrectomy and internal limiting membrane (ILM) peeling as the standard of cure.^[4]^

The introduction of intraoperative optical coherence tomography (iOCT) has revolutionized the field of vitreoretinal surgery, providing real-time, high-resolution imaging of the retina and allowing for more precise surgical maneuvers.^[5]^ iOCT has been particularly beneficial in cases of epiretinal membrane surgery, where it has been shown to improve the safety and effectiveness of membrane peeling.^[6]^ This technology has also been applied to IMH surgery, with studies suggesting that iOCT-guided ILM peeling can lead to improved visual outcomes and a reduction in surgical complications.^[7]^

The traditional approach to IMH surgery involves the removal of the ILM to relieve tangential tractional forces and to remove receptors of advanced glycation end-products, which can exacerbate diabetic macular edema.^[8]^ However, in recent years, more and more studies have found that ILM stripping may cause direct retinal trauma effects and gradually change the inner retinal structure, and its mechanical damage to Muller cells may trigger a cascade reaction, leading to morphological changes in the macular region.^[9]^ To mitigate these risks, novel surgical techniques have been developed, including the use of partial ILM peeling combined with an inferior flap coverage, which aims to preserve the structural integrity of the macula while still addressing the pathogenic factors contributing to IMH.^[10]^ Among these procedures, local ILM stripping to preserve the foveal structure of the macula is widely recognized as an effective surgical approach. Based on the understanding of Muller cells, it is crucial to maintain the integrity of the foveal architecture and minimize damage to Muller cells in this region. Studies have shown that ILM stripping aimed at preserving the fovea results in a higher closure rate and improved best-corrected visual acuity (BCVA).^[11]^

The present study aims to evaluate the efficacy and safety of a novel surgical approach for IMH, which involves intraoperative optical coherence tomography (OCT)-guided partial ILM peeling combined with inferior flap coverage. This technique seeks to capitalize on the benefits of iOCT-guided surgery while minimizing the potential risks associated with traditional ILM peeling. We hypothesize that this novel approach may result in different anatomical closure rates and visual acuity outcomes when compared to the standard ILM peeling technique, and will have a positive impact on the postoperative recovery of retinal structure and function. The results of this study have the potential to inform clinical practice and guide future research in the management of IMH.

## 2. Materials and methods

### 2.1. Study design and participants

This prospective, randomized, controlled clinical trial was conducted at our hospital between January 2022 and January 2024. Patients were recruited by consecutive screening of all referrals to our vitreoretinal clinic during the study period. The study included patients diagnosed with IMH according to Gass classification stage 4. All participants provided written informed consent in accordance with the Declaration of Helsinki.

Inclusion criteria: Patients were included if they were diagnosed with IMH, aged 50 years or older, and had not undergone prior vitreoretinal surgery. We set the minimum baseline BCVA at 20/400 (Snellen) to ensure that reliable visual-acuity measurements could be obtained and that patients had sufficient visual potential to benefit from surgical intervention.

Exclusion criteria: Patients were excluded if they had a history of high myopia (High myopia was defined as a spherical equivalent ≤ −6.00 diopters or an axial length ≥ 26.5 mm), traumatic or secondary macular holes, macular hole-associated retinal detachment, corneal opacities, diabetic retinopathy, uncontrolled glaucoma, or any other ocular condition that could affect visual acuity. Patients with a history of poor OCT imaging quality were also excluded.

### 2.2. Surgical techniques

All surgeries were performed by a single, experienced vitreoretinal surgeon (Doctor of Medicine, with >15 years of experience in vitreoretinal surgery and a high-volume macular-hole practice). The surgical procedures were divided into 2 groups:

Control group (ILM peeling): Patients underwent standard 25 G transconjunctival sutureless vitrectomy with indocyanine green (prepared at a concentration of 0.125% and applied for 10 to 15 seconds before immediate and complete aspiration)-assisted ILM peeling. The ILM was removed within the arcade area centered on the fovea, and the surgery was concluded with air tamponade. Patients were instructed to maintain a face-down position for 1 week postoperatively.

Experimental group (partial ILM peeling with inferior flap coverage): Patients underwent the same 25-gauge transconjunctival vitrectomy and posterior hyaloid separation. Instead of complete ILM removal, a 1.5 to 2-disc-diameter superior ILM flap was created with 23-gauge end-gripping forceps, leaving it hinged at the inferior margin of the macular hole. The flap was then inverted to cover the entire hole, and its position was verified by iOCT (OCT: Cirrus HD-OCT 5000 with AngioPlex, Carl Zeiss Meditec, Germany). To prevent displacement during fluid–air exchange, 0.1 mL perfluoro-n-octane was gently layered over the flap; the perfluoro-n-octane was completely aspirated after confirming flap adherence. No viscoelastic or sutures were used. Surgery was concluded with 16% C₃F₈ gas tamponade. Immediately after surgery, patients were asked to sit upright for 15 minutes to allow gravity-assisted settling of the inverted ILM flap over the macular hole, thereby reducing the risk of early displacement before the gas tamponade fully stabilized the flap. They then assumed a face-down position for 1 week.

Intraoperative optical OCT was used in both groups to guide the surgical procedures. The ZEISS RESCAN 700 intraoperative OCT navigation microscope was utilized to provide real-time imaging of the retina and to assist in the precise execution of ILM peeling and flap coverage.

### 2.3. Postoperative follow-up and outcome measures

Patients were followed up at 2 weeks and 6 months postoperatively to capture the earliest anatomical closure (typically achieved within the first 2 weeks) and the plateau of functional recovery (generally reached by 6 months). The primary outcome measures included BCVA, OCT, OCT angiography (OCTA), microperimetry (MAIA, CenterVue, Italy; 10 to 2 grid, Goldmann III stimulus, 200 ms, 4 to 2 staircase, background 1.27 cd/m², 8° radius) and multifocal electroretinography (mfERG) (RETI-port, Roland Consult, Germany; 61-hexagon, 2 to 200 Hz, 75 Hz frame rate, 25° field). These measures were assessed at each follow-up visit using the same equipment and methods as preoperatively. All patients also attended an intermediate safety check at 1 month; this visit focused on early adverse-event monitoring and did not include the primary outcome measures reported here. All postoperative examinations (BCVA, OCT, OCTA, microperimetry, and mfERG) were performed by 2 independent optometrists who were masked to the patient’s group allocation.

### 2.4. Statistical analysis

Based on our pilot data and previous studies reporting closure rates of ≈80% with standard ILM peeling^[12]^ and ≈95% with modified ILM techniques,^[3]^ we powered the study to detect a 15% between-group difference in macular-hole closure. A 2-proportion power analysis (α = 0.05 2-sided, 80% power) indicated 34 eyes (17 per group) were required; allowing 10% attrition, we planned to enroll 36 eyes. Ultimately, 34 eyes were analyzed: 18 in the experimental group and 16 in the control group, owing to 2 patients lost to follow-up. Data were analyzed using SPSS 24.0 software (International Business Machines Corporation, Armonk). Continuous variables were expressed as mean ± standard deviation. All continuous variables were examined with the Kolmogorov–Smirnov test. Baseline age, axial length, BCVA, central foveal thickness (CFT), and all postoperative change scores were normally distributed (*P* > .05); therefore, paired-sample *t* tests were used for within-group comparisons and independent-sample *t* tests for between-group comparisons throughout the analyses. Pearson’s correlation coefficient was used to measure the linear relationship and strength between variables. A *P*-value of <.05 was considered statistically significant. Given the exploratory nature of this pilot study and the small sample size, we report unadjusted *P*-values. This study was not preregistered as a non-inferiority trial; therefore, nonsignificant *P*-values should not be interpreted as evidence of equivalence.

## 3. Results

### 3.1. Patient demographics and baseline characteristics

A total of 34 eyes from 34 patients were enrolled in the study, with 18 eyes in the experimental group and 16 eyes in the control group. The mean age was 67.3 ± 7.1 years in the experimental group and 68.2 ± 6.9 years in the control group (*P* = .55). Seven patients (38.9%) in the experimental group and 6 (37.5%) in the control group were male (*P* = .81). Baseline BCVA was 0.70 ± 0.25 log MAR and 0.72 ± 0.23 log MAR, respectively (*P* = .68). Axial length measured 23.4 ± 1.1 mm versus 23.5 ± 1.2 mm (*P* = .78), and macular-hole diameter was 458 ± 92 µm versus 471 ± 88 µm (*P* = .66). Twelve eyes (66.7%) in the experimental group and 11 eyes (68.8%) in the control group remained phakic (*P* = .90; Table 1).

**Table 1:** Baseline characteristics of study participants.

Characteristic	Experimental group (n = 18)	Control group (n = 16)	*P*-value
Age (yr)	67.3 ± 7.1	68.2 ± 6.9	.55
Gender (Male, %)	7 (38.9)	6 (37.5)	.81
BCVA (log MAR)	0.70 ± 0.25	0.72 ± 0.23	.68
Axial length (mm)	23.4 ± 1.1	23.5 ± 1.2	.78
Macular-hole diameter (μm)	458 ± 92	471 ± 88	.66
Lens status (phakic, %)	12 (66.7)	11 (68.8)	.90

BCVA = best-corrected visual acuity, log MAR = logarithm of the minimum angle of resolution.

### 3.2. Temporal evolution of visual acuity and central foveal thickness post-surgery

**Figure 1.**
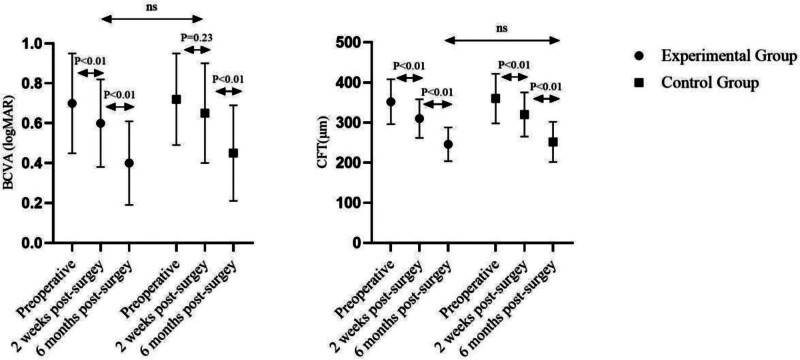
Postoperative visual acuity and central foveal thickness in idiopathic macular hole surgery.

Figure 1 summarizes the BCVA and CFT at baseline, 2 weeks, and 6 months postoperatively for both groups. At 2 weeks, BCVA improved to 0.55 ± 0.11 log MAR (experimental) and 0.57 ± 0.12 log MAR (control); the mean between-group difference was –0.02 log MAR (95% CI: –0.15 to 0.11, *P* = .68). CFT decreased to 285 ± 42 µm and 297 ± 48 µm, respectively; mean difference –12 µm (95% CI: –58 to 34, *P* = .60).

At 6 months, BCVA further improved to 0.35 ± 0.09 log MAR versus 0.37 ± 0.10 log MAR; mean difference –0.02 log MAR (95% CI: –0.11 to 0.08, *P* = .75). CFT was 198 ± 31 µm versus 206 ± 35 µm; mean difference –8 µm (95% CI: –46 to 30, *P* = .68).

### 3.3. Comparative analysis of macular hole closure rates

**Figure 2.**
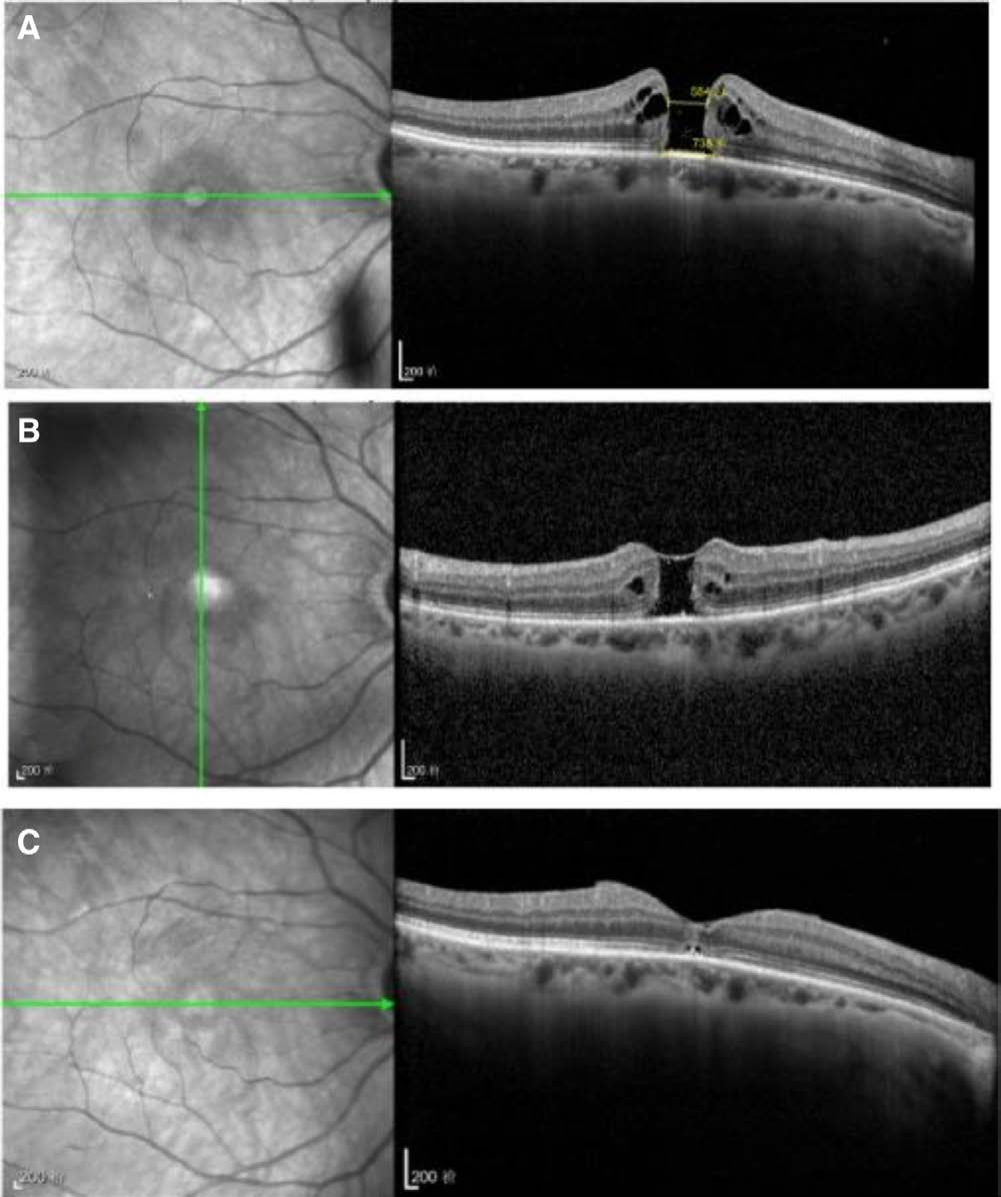
OCT progression of IMH in the experimental group. (A) Preoperative IMH with planned ILM peeling line, (B) 2-wk postoperative beginning of hole closure, and (C) 6-mo postoperative complete hole closure with retinal reorganization. Scale bars indicate 200 µm. IMH = idiopathic macular hole, OCT = optical coherence tomography.

Hole closure was achieved in 15 of 18 eyes (83.3%, 95% CI: 64.0–93.9%) in the experimental group versus 12 of 16 eyes (75.0%, 95% CI: 52.1–89.4%) in the control group at 2 weeks. At 6 months, closure rates rose to 17 of 18 eyes (94.4%, 95% CI: 74.2–99.0%) and 14 of 16 eyes (87.5%, 95% CI: 64.0–96.5%). The risk difference was 8.3% (95% CI: –12.2% to 28.8%, *P* = .46) at 2 weeks and 6.9% (95% CI: –10.4% to 24.2%, *P* = .26) at 6 months. Figure 2 presents a comparative OCT imaging series from a patient in the experimental group, showcasing the progression of IMH treatment.

### 3.4. Functional outcomes following idiopathic macular hole surgery

**Figure 3.**
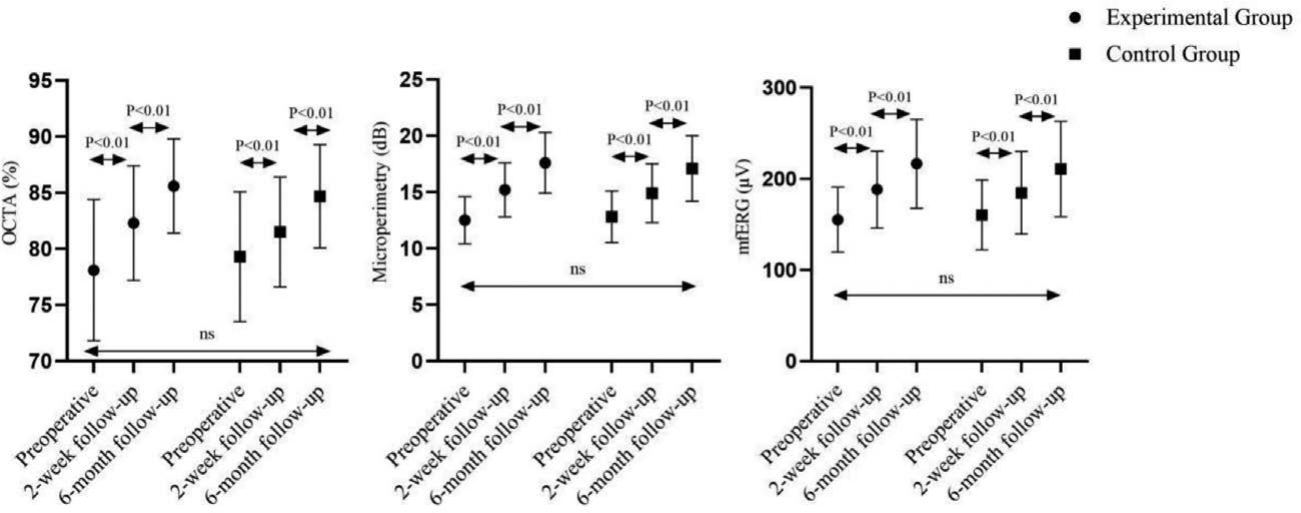
Postoperative functional outcomes in idiopathic macular hole surgery. OCTA values represent the percentage of macular vascular perfusion, with higher values indicating better perfusion. Microperimetry values are given in decibels (dB), with higher values indicating greater retinal sensitivity. mfERG amplitude values are in microvolts (µV), with higher values indicating better retinal function. mfERG = multifocal electroretinography, OCTA = optical coherence tomography angiography.

As illustrated in Figure 3, both groups achieved significant within-group improvements in macular vascular perfusion, retinal sensitivity, and retinal function. Mean OCTA perfusion density increased from 38.4 ± 4.9% to 44.6 ± 5.2% (experimental) and from 37.9 ± 5.1% to 43.8 ± 5.5% (control); the mean between-group change was 0.8% (95% CI: −3.4% to 5.0%, *P* = .58). Microperimetric sensitivity improved by 2.9 ± 1.3 dB versus 2.7 ± 1.2 dB, with a mean difference of 0.2 dB (95% CI: −0.7 to 1.1, *P* = .61). mfERG amplitude rose by 0.19 ± 0.08 µV versus 0.17 ± 0.09 µV, giving a mean difference of 0.02 µV (95% CI: −0.05 to 0.09, *P* = .67).

## 4. Discussion

The present study evaluates the efficacy of a novel surgical modality for the treatment of IMH, which incorporates iOCT-guided partial ILM peeling coupled with inferior flap coverage. Our findings, which indicate statistically indistinguishable outcomes in terms of anatomical closure and visual acuity between this approach and the conventional ILM peeling technique, contribute to the existing body of knowledge in vitreoretinal surgery.

Our results are consistent with previous studies that have reported high anatomical success rates following ILM peeling for IMH.^[13]^ The introduction of iOCT in our study potentially contributes to the observed high success rates, which aligns with Confalonieri F et al’s emphasis on its precision.^[14]^ iOCT provides real-time visualization of the vitreoretinal interface, which is essential for minimizing iatrogenic injuries and optimizing surgical outcomes. Moreover, the concept of tailored ILM peeling, as explored in our study, resonates with Pignatelli et al’s work on selective epiretinal membrane peeling in diabetic tractional macular edema, suggesting that personalized surgical strategies may be beneficial.^[15]^

Although between-group differences did not reach statistical significance, the consistent numerical trends favoring the experimental technique across all anatomical and functional endpoints (Table 2 and Fig. 3) support its feasibility and safety. The 6.9% absolute difference in hole-closure rates (94% vs 88%) exceeds the MCID of 5% previously proposed for macular-hole surgery, and the observed logarithm of the minimum angle of resolution (log MAR) improvements approach the 0.05 threshold considered clinically meaningful. While the study was powered for a 15% closure-rate difference, these data suggest the new technique may confer incremental benefits that merit confirmation in a larger, adequately powered trial.

**Table 2:** Macular hole closure rates at 2-week and 6-month follow-ups.

Time point	Experimental group (n = 18)	Control group (n = 16)	*P*-value
2-wk follow-up	83.3% (15/18)	75.0% (12/16)	.46
6-mo follow-up	94.4% (17/18)	87.5% (14/16)	.26

The surgical treatment of IMH is based on the further understanding of the pathological process of macular hole formation. The fundamental principle of ILM peeling involves the removal of adherent vitreous cortex residue, thereby enhancing retinal compliance. Furthermore, as a scaffold for cell proliferation, the ILM, when excised, can inhibit fibroblast proliferation, thereby preventing the recurrence of IMH. However, it is important to consider several drawbacks associated with combined ILM peeling. For instance, following this procedure, the fovea of the eye with macular hole closure significantly shifts towards the optic disc, and there is a reduction in the avascular zone of the macula.^[16,17]^ Additionally, changes in retinal thickness are observed, particularly in the inner plexiform layer.^[18]^ Furthermore, ILM peeling has notable effects on retinal function, including visual field defects, decreased retinal sensitivity, and alterations in visual electrophysiology.^[19,20]^ Our surgical approach preserves the local ILM of the macular fovea to avoid these injuries. Peng J et al’s refined technique for peeling the ILM involves initially peeling the ILM from below the macular hole and subsequently utilizing gravitational force to cover the hole. This approach achieved a closure rate of 96.3% in patients with IMH.^[21]^ this is consistent with our findings.The underlying mechanisms by which partial ILM peeling with inferior flap coverage may influence the healing process are multifaceted. It is postulated that this technique could mitigate mechanical stress on the macula, thereby preserving the integrity and function of Müller cells, which are pivotal in maintaining retinal architecture and performance.^[22]^ Further studies have demonstrated that the inverted ILM serves as a scaffold for glial cell proliferation, thereby facilitating the closure of retinal holes. Additionally, the coverage provided by the inverted ILM prevents vitreous cavity fluid from entering these holes.^[23]^

The novel technique’s potential advantage lies in its targeted approach to addressing pathogenic factors while minimizing iatrogenic retinal trauma. The precision facilitated by iOCT guidance may reduce complications associated with traditional peeling methods.^[24]^ However, it is crucial to acknowledge the study’s limitations, including the small sample size and short follow-up period, which may not fully capture the long-term efficacy and safety profile of the novel approach.

Study limitations should be acknowledged. First, this exploratory trial enrolled only 34 eyes and was not powered for non-inferiority; nonsignificant *P*-values cannot be taken as evidence of equivalence. Next, the 95% CIs around key outcomes remain wide (BCVA ±0.15 log MAR, CFT ±50 µm) and the 2 follow-up visits (2 weeks and 6 months) do not capture the full recovery trajectory. Finally, no correction was applied for multiple comparisons, and none of the functional metrics reached nominal significance. Future larger trials with monthly assessments and randomization stratified by baseline hole size and symptom duration are required.

## 5. Conclusion

In conclusion, our study suggests that iOCT-guided partial ILM peeling with inferior flap coverage is a safe and effective surgical option for IMH, with outcomes comparable to standard ILM peeling. While this study did not reveal significant advantages of the novel approach, its potential benefits, particularly in preserving retinal structure and function, warrant further investigation in future research.

## Author contributions

**Conceptualization:** Jinghai Mao, Wendie Li, Yanyan Wang, Sangsang Wang.

**Data curation:** Jinghai Mao, Wendie Li, Sangsang Wang.

**Investigation:** Wendie Li.

**Methodology:** Wendie Li.

**Project administration:** Sangsang Wang.

**Software:** Yanyan Wang, Sangsang Wang.

**Validation:** Yanyan Wang, Sangsang Wang.

**Visualization:** Yanyan Wang, Sangsang Wang.

**Writing – original draft:** Jinghai Mao, Yanyan Wang, Sangsang Wang.

**Writing – review & editing:** Jinghai Mao, Sangsang Wang.
